# Lifetime inbreeding depression in a leaf beetle

**DOI:** 10.1002/ece3.4205

**Published:** 2018-06-22

**Authors:** Thorben Müller, Tabea Dagmar Lamprecht, Karin Schrieber

**Affiliations:** ^1^ Department of Chemical Ecology Bielefeld University Bielefeld Germany; ^2^ Martin‐Luther‐University Halle‐Wittenberg Institute of Biology, Geobotany and Botanical Garden Halle (Saale) Germany

**Keywords:** development, inbreeding avoidance, mating system, *Phaedon cochleariae*, reproduction, second male sperm precedence

## Abstract

Ongoing habitat loss and fragmentation result in rapid population size reductions, which can increase the levels of inbreeding. Consequently, many species are threatened by inbreeding depression, a loss of individual fitness following the mating of close relatives. Here, we investigated inbreeding effects on fitness‐related traits throughout the lifetime of the mustard leaf beetle (*Phaedon cochleariae*) and mechanisms for the avoidance of inbreeding. Previously, we found that these beetles have family‐specific cuticular hydrocarbon profiles, which are likely not used as recognition cue for precopulatory inbreeding avoidance. Thus, we examined whether adult beetles show postcopulatory inbreeding avoidance instead. For this purpose, we determined the larval hatching rate of eggs laid by females mated sequentially with two nonsiblings, two siblings, a nonsibling, and a sibling or *vice versa*. The beetles suffered from inbreeding depression throughout their entire ontogeny, as evinced by a prolonged larval development, a decreased larval and adult survival and a decreased reproductive output of inbred compared to outbred individuals. The highest larval hatching rates were detected when females were mated with two nonsiblings or first with a sibling and second with a nonsibling. Significantly lower hatching rates were measured in the treatments with a sibling as second male. Thus, the results do not support the existence of postcopulatory inbreeding avoidance in *P. cochleariae*, but revealed evidence for second male sperm precedence. Consequently, an alternative strategy to avoid inbreeding costs might exist in this beetle, such as a polyandrous mating system, potentially coupled with a specific dispersal behavior.

## 1 INTRODUCTION

In the course of ongoing global change, an ever‐increasing number of species is threatened by habitat loss and fragmentation (Haddad et al., 2015; Murphy, Battocletti, Tinghitella, Wimp, & Ries, 2016; Swift & Hannon, 2010). This habitat change can entail a severe reduction in the size of natural populations and simultaneously increase the degree of isolation among them (Agnarsson, Aviles, & Maddison, 2013; Bates, Sadler, et al., 2014). Strong population declines are typically associated with genetic drift and a loss of genetic variation, which can be amplified by restricted gene flow among populations, if habitats exhibit low connectivity (Aguilar, Quesada, Ashworth, Herrerias‐Diego, & Lobo, 2008; Murphy et al., 2016). Such demographic events can reduce the viability of populations, as they increase the likelihood of matings among closely related individuals (i.e., inbreeding), which can have highly detrimental effects on fitness (Banks, Piggott, Stow, & Taylor, 2007; Lane, Forrest, & Willis, 2011). Hence, a broad understanding of these inbreeding effects on the life history of different species and the occurrence of inbreeding avoidance mechanisms is important to predict and manage the rapid biodiversity declines under global change (Schmitz et al., 2014).

Inbreeding can induce manifold detriments in affected organisms, which typically culminate in reduced survival and/or reproductive success of inbred relative to outbred individuals (Fox, Scheibly, Smith, & Wallin, 2007; Harano, 2011; Lihoreau, Zimmer, & Rivault, 2007). This loss of individual fitness following inbreeding is referred to as inbreeding depression. Inbreeding depression arises from an increase in genome‐wide homozygosity in the offspring generation, which results in the increased phenotypic expression of recessive deleterious mutations and the reduced expression of heterozygote advantage (Charlesworth & Willis, 2009; Keller & Waller, 2002). Inbreeding depression can become evident in the offspring of only one generation inbreeding (Müller & Müller, 2016) or after several generations inbreeding (Bilde, Maklakov, & Schilling, 2007). Moreover, inbreeding can also influence traits not closely linked to fitness, such as the chemical (Menzel, Radke, & Foitzik, 2016) or behavioral phenotype of insects (Müller & Juškauskas, 2018; Pilakouta & Smiseth, 2017; Richardson & Smiseth, 2017).

Given the high inbreeding costs (inbreeding depression) and the comparably low probability to benefit from inbreeding (reproductive assurance in the absence of non‐related mating partners and inclusive fitness benefits; Peer & Taborsky, 2005; Kokko & Ots, 2006), many animals evolved mechanisms to avoid the mating with close relatives (Pusey & Wolf, 1996). These avoidance mechanisms come into action either before (precopulatory, e.g., Liu, Tu, He, Chen, & Xue, 2014) or after (postcopulatory, e.g., Simmons, Beveridge, Wedell, & Tregenza, 2006; Bretman, Newcombe, & Tregenza, 2009) inbreeding events. Precopulatory mechanisms base on the discrimination of kins as mating partners (Lihoreau & Rivault, 2009; Metzger, Bernstein, Hoffmeister, & Desouhant, 2010; Whitehorn, Tinsley, & Goulson, 2009). Postcopulatory mechanism can rest, for example, on kin sperm discrimination (Bretman et al., 2009; Welke & Schneider, 2009), that is, a lowered transfer of sperm if females mate with closely related males (Lewis & Wedell, 2009). Family‐specific cuticular hydrocarbon (CHC) (Lihoreau & Rivault, 2009) or pheromone patterns (Herzner, Schmitt, Heckel, Schreier, & Strohm, 2006) partly serve as recognition cues on which inbreeding avoidance mechanisms are based on (Lihoreau & Rivault, 2009; Thomas & Simmons, 2011). However, pre‐ or postcopulatory kin discrimination as inbreeding avoidance mechanisms is not necessarily expressed in all species suffering from inbreeding depression (Bouchebti, Durier, Pasquaretta, Rivault, & Lihoreau, 2016; Edvardsson, Rodriguez‐Munoz, & Tregenza, 2008). As alternative or in combination, the costs of inbreeding can be reduced by a polyandrous mating system (Bayoumy, Michaud, & Bain, 2015; Duthie, Bocedi, & Reid, 2016; Tregenza & Wedell, 2002). Moreover, a specific dispersal strategy can lead to inbreeding avoidance by preventing the encountering of closely related individuals (Pusey & Wolf, 1996).

**Figure 1:**
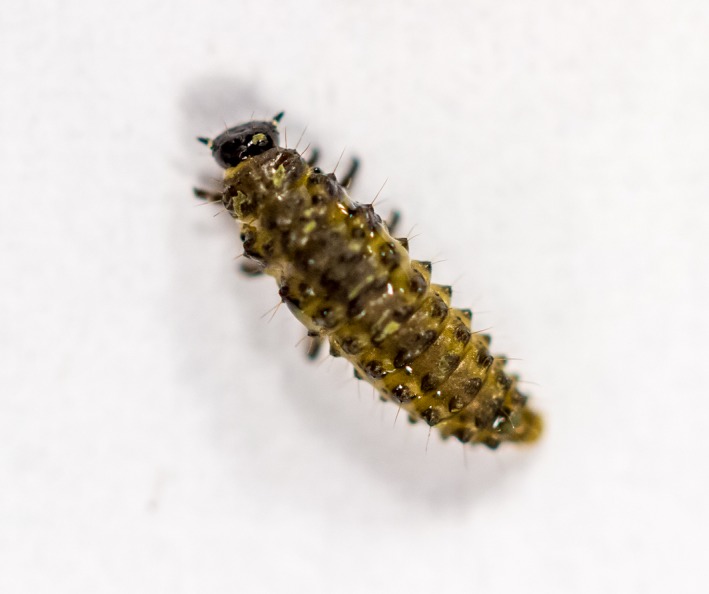
*Phaedon cochleariae*, mustard leaf beetle (Coleoptera: Chrysomelidae), larvae in the third (last) larval stage (photograph was taken by T. Müller, F. Bien, and C. Engelbrecht)

In this study, we investigated the effects of inbreeding over the lifetime of *Phaedon cochleariae* F. (Coleoptera: Chrysomelidae, Figure 1). We determined the larval development time, larval and adult survival, adult body mass, and reproductive output of outbred *versus* inbred mustard leaf beetles. Moreover, we conducted a mating assay in order to test whether postcopulatory inbreeding avoidance mechanisms occur in our study species. We sequentially mated females with two nonsiblings, two siblings, a nonsibling and a sibling, and a sibling and a nonsibling. The reproductive output of these distinct mating combinations was detected by determining the larval hatching rate of eggs laid by the females.

In a previous study on *P. cochleariae*, we disclosed that the larval hatching rate of inbred offspring is decreased in comparison with outbred offspring (Müller & Müller, 2016). Thus, this beetle species suffers from inbreeding depression and we consequently expected negative inbreeding effects on other fitness‐related traits throughout its ontogeny. Moreover, the CHC profile, which is decisive for mate choice in *P. cochleariae* (Geiselhardt, Otte, & Hilker, 2012) and other insect species (Howard & Blomquist, 2005), differs between families (Müller & Müller, 2016). Although CHCs can function as mechanism to discriminate between closely related and unrelated individuals (Weddle, Hunt, & Sakaluk, 2013), the family‐specific CHC patterns in *P. cochleariae* do not mediate precopulatory inbreeding avoidance by a faster mating of nonsiblings compared to siblings (Müller & Müller, 2016). Thus, family‐specific CHC profiles might serve as a recognition cue for postcopulatory inbreeding avoidance. Here, we specifically tested whether such postcopulatory inbreeding avoidance mechanisms exist in *P. cochleariae*. In detail, we hypothesized a lower hatching rate of larvae from eggs laid by females that were sequentially mated with two siblings compared to a mating with two nonsiblings or a nonsibling and a sibling (or *vice versa*). Such patterns would indicate the existence of sibling sperm recognition and discrimination (Tregenza & Wedell, 2002).

## 2 MATERIALS AND METHODS

### 2.1 Study organisms and rearing

*Phaedon cochleariae* individuals of our laboratory strain descended from different regions in Germany, that is, side arms of the river Main, Botanical Garden of Berlin‐Dahlem and the south of Bielefeld along the Furlbach. This mixed strain, which consisted of 500–600 individuals, was reared for approximately 25 generations under laboratory conditions, where individuals mated randomly. Every year, 100–150 wild *P. cochleariae* individuals were collected at the Bielefeld location and were integrated into the strain in order to refresh the gene pool. Beetles were reared in separate ventilated plastic boxes (20 × 20 × 6.5 cm) in a climate cabinet (20°C, 16:8 hr light:dark, 65% relative humidity) with a density of about 100–200 individuals per box and a nearly balanced sex ratio. The beetles were fed ad libitum with leaves of 8–10 weeks old *Brassica rapa* L. ssp. *pekinensis* var. Michihili plants (seeds received from Kiepenkerl; Bruno Nebelung GmbH, Konken, Germany). Plants were grown in a greenhouse (16:8 hr light:dark, 60% relative humidity) in pots (12 cm diameter) filled with composted soil.

### 2.2 Experiment 1: measurements of larval development time, larval and adult survival, adult body mass, and reproductive output

Pupae originating from three different rearing boxes were collected and each pupa and later each young adult was reared separately in one small petri dish (5.5 cm diameter). Thus, unintended mating combinations and sibling matings were prevented prior to the experiment. After adult emergence and sex determination, pairs of one female and one male, which descended from different rearing boxes, were transferred into small petri dishes (one pair per petri dish). These individuals served as parental (P) generation. In total, six different breeding pairs (corresponding to six families) were used. Eggs laid by the females were separately collected and larvae (F1 generation) were reared in large petri dishes (9 cm diameter) until pupation. Pupae were separated and the emerging F1 adults were again reared in a density of one individual per small petri dish. The F1 generation was used to examine the effects of inbreeding on various fitness‐related traits over the lifetime of *P. cochleariae*. For this purpose, we performed experimental outbreeding and inbreeding with the F1 generation. Here, we set up pairs of one female and one male, which were either nonsiblings (i.e., outbreeding) or siblings (i.e., inbreeding) for each of the six families. We used two to four pairs per family x breeding treatment combination, depending on the availability of *P. cochleariae* individuals. All pairs were separately reared in small petri dishes for 14 days. During this time interval, each pair mated repeatedly and all females laid eggs. Subsequently, we collected the eggs produced by nonsibling pairs (i.e., outbred offspring) and sibling pairs (i.e., inbred offspring) and transferred them to new large petri dishes (one separate dish for eggs of each mating pair). Based on the performed breeding scheme, the parents of outbred offspring were neither full nor half siblings nor cousins and the parents of inbred offspring were all full siblings.

After hatching of the outbred and inbred larvae (F2 generation), the days until adult emergence were counted (i.e., development time). During that time, all larvae descending from identical parents were captured in one large petri dish with up to 18 individuals. Moreover, we determined the proportion of surviving larva until pupation (larval survival) as well as the proportion of viable adults directly after adult emergence (adult survival) per petri dish. Adult beetles were then transferred into new small petri dishes (one beetle per petri dish). At day 8–10 of adult life, the body mass of virgin males and females was determined, using a microbalance (ME36S, accuracy 0.001 mg; Sartorius AG, Göttingen, Germany). Afterwards, outbred and inbred females were mated with an outbred male of a different family. The number of eggs laid per female was counted over a period of 4 days. At the first day of counting, the females were 11–16 days old (predictor variable age). All F1 and F2 beetles were reared ad libitum on middle‐aged cabbage leaf disks, which were replaced every to every other day. All petri dishes were lined with a moistened filter paper to prevent desiccation.

### 2.3 Experiment 2: effects of nonsibling and sibling mating partner order on larval hatching rate

In experiment 2, we investigated whether postcopulatory inbreeding avoidance mechanisms occur in *P. cochleariae*. For this purpose, we set up four mating assay treatments, using either nonsiblings (i.e., outbreeding) or siblings (i.e., inbreeding) from the F1 generation (see previous section) as mating partners. In the first treatment group, females were sequentially mated with two nonsibling males, in the second group first with a nonsibling and second with a sibling, in the third group first with a sibling and second with a nonsibling and in the fourth group sequentially with two sibling males. We used beetles of all families for each mating combination. For each treatment, F1 females and males were separately captured in small petri dishes until day 10 of adult life. Thereafter, the first virgin male was added to the petri dish of the virgin female at about 10.00 a.m. After 6 hr, this first male was removed and after additional 18 hr the second virgin male was added for 6 hr from 10.00 a.m. to 16.00 p.m. To prevent confounding effects of daytime on mating success (matings might be avoided in the evening hours, as beetles are mainly active from 10 a.m. to 16 p.m.), we did not add the second male at the same day after 16.00 p.m. We checked whether beetles mated during the time spent together in the petri dish. Three, 5 and 7 days after the last mating occurred, all eggs laid by a specific female in 24 hr were counted and removed from the petri dish. We determined the proportion of larvae hatched from all eggs laid per female individual (i.e., hatching rate). Beetles for experiment 2 were reared in the same way as for experiment 1 (see above).

### 2.4 Statistical analyses

All data on the performance of *P. cochleariae* from both experiments were statistically analyzed with mixed effects models (R‐package: *lme4*, Bates, Maechler, Bolker, & Walker, 2014) in R version 3.2.3 (R Development Core Team, 2015). For responses with normal error distribution, we used linear mixed effects models (LMM), whereas for responses with Poisson or binomial error distribution, we used generalized linear mixed effects models (GLMM).

The model for the response development time (GLMM, Poisson) from experiment 1 comprised the fixed effects of breeding treatment and the random effects of family in P‐generation and petri dish nested within family. The models for the responses larval survival (GLMM, binomial) and adult survival (GLMM, binomial) from experiment 1 included the fixed effect of breeding treatment and the random effect of family in P‐generation. The model for the response body mass (LMM, Gaussian, log‐transformed) from experiment 1 comprised the fixed effects of breeding treatment, sex (covariate) and the interaction among these factors as well as the random effect of family in P‐generation. The model for the response egg number (GLMM, Poisson) from experiment 1 included the fixed effects of breeding treatment, female age (covariate) and their interaction as well as the random effect of family in P‐generation. The model for the response larval hatching (GLMM, binomial) from experiment 2 included the fixed effects of mating treatment and the random effect family in P‐generation.

All of the described models were fitted with a maximum likelihood approach. After assuring that models exhibit variance homogeneity and normal distribution of residuals by the means of visual inspection, we applied stepwise backward model selection to obtain the minimal adequate model. Here, we removed fixed effect terms with *p* > 0.05 based on chi‐square likelihood ratio tests (R‐package: *MASS*, Venables & Ripley, 2000). In case a minimal adequate mixed model included fixed effect factors with more than two levels (mating treatment from experiment 2), we additionally performed multiple comparisons (Tukey post hoc tests) of all factor levels on the respective model (R‐package: *multcomp*, Hothorn, Bretz, & Westfall, 2008). For illustration of the effects of breeding treatment (experiment 1) and mating treatment (experiment 2), we generated box‐whisker plots, which additionally included least square means with their standard errors extracted from the respective minimal adequate mixed effects models (R‐package: *lsmeans*, Lenth, 2016). In contrast to raw data means and their standard errors, these model estimates account for the specific error distribution of the response, for the effects of covariates as well as for random effects.

## 3 RESULTS

### 3.1 Inbreeding effects on fitness‐related traits

**Figure 2:**
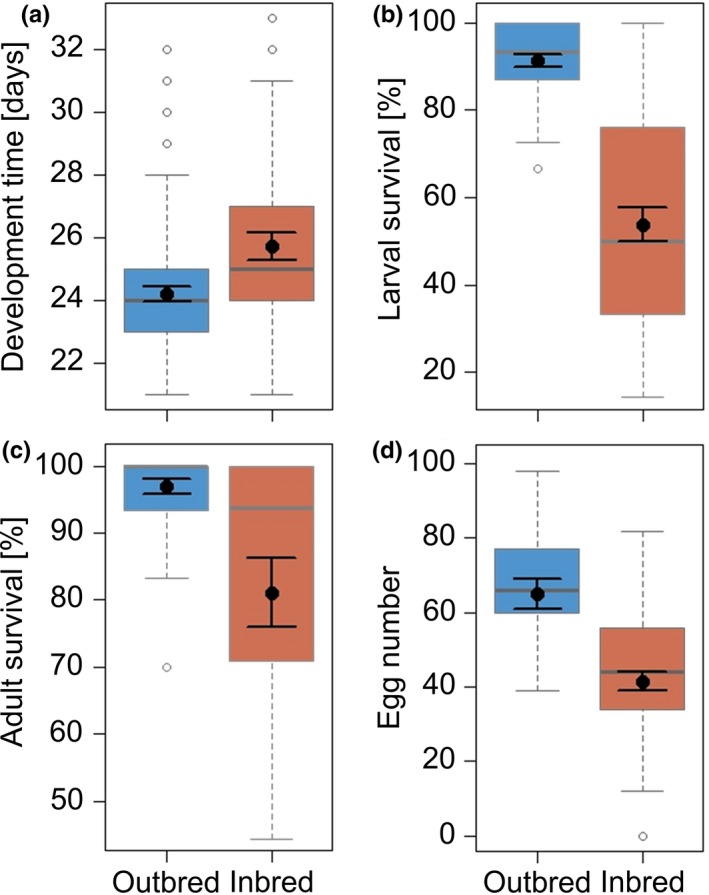
Effects of breeding treatment (outbred *versus* inbred) on (a) development time (*N*_Outbred_ = 437, *N*_Inbred_ = 131), (b) larval survival (*N*_Outbred_ = 37, *N*_Inbred_ = 28), (c) adult survival directly after emergence (*N*_Outbred_ = 37, *N*_Inbred_ = 28), and d) the egg number laid over a period of 4 days per female (*N*_Outbred_ = 25, *N*_Inbred_ = 28) of *Phaedon cochleariae*. The box‐whisker plots show the following statistics: medians (solid gray lines), interquartile range (boxes), 1.5 * lower/upper quartile (whiskers), outliers (white dots), and least square means with their standard error (black dots with error bars) extracted from the minimal adequate (G)LMM

The larval development time, larval survival rate, viability of freshly hatched adults, and the reproductive output of *P. cochleariae* were significantly affected by inbreeding (Figure 2, Tables 1, 2). The development time of inbred larvae was on average 1.5 days prolonged compared to outbred larvae (Figure 2a). The larval survival rate until adulthood was 37.6% lower in inbred (53.8% larvae survived) than in outbred individuals (91.4% larvae survived; Figure 2b). Moreover, only 81.5% of the inbred beetles were viable after adult emergence, compared to 96.9% of the outbred beetles (Figure 2c). Likewise, the number of eggs laid by inbred females was 36.2% lower than in outbred females (Figure 2d). Adult body mass was significantly affected by the covariate sex, with females being heavier than males (Table 1).

**Table 1:** Effects of breeding treatment [outbred (O) *versus* inbred (I)], covariates, and their interaction on development time (*N*_O_ = 437, *N*_I_ = 131), larval survival (*N*_O_ = 37, *N*_I_ = 28), adult survival (*N*_O_ = 37, *N*_I_ = 28), adult body mass (*N*_O_ = 20–30, *N*_I_ = 15–17), and egg number (*N*_O_ = 25, *N*_I_ = 28) of *Phaedon cochleariae* from experiment 1 (E1), as well as effect of mating treatment [male order: nonsibling, nonsibling (N/N) *versus* nonsibling, sibling (N/S) *versus* sibling, nonsibling (S/N) *versus* sibling, sibling (S/S)] on larval hatching rate (*N*_N/N_ = 24, *N*_N/S_ = 25, *N*_S/N_ = 25, *N*_S/S_ = 25) of *Phaedon cochleariae* from experiment 2 (E2)

Responses:	Development time (E1)		Larval survival (E1)		Adult survival (E1)		Body mass (E1)		Egg number (E1)		Larval hatching (E2)	
	GLMM (Poisson)		GLMM (Binomial)		GLMM (Binomial)		LMM (Gaussian)		GLMM (Poisson)		GLMM (Binomial)	
Fixed effects:	*X²*	*p*	*X²*	*p*	*X²*	*p*	*X²*	*p*	*X²*	*p*	*X²*	*p*
Breeding treatment	9.350	**0.002**	127.950	**<0.001**	30.774	**<0.001**	3.305	0.069	130.980	**<0.001**	Not tested	
Sex	Not tested		Not tested		Not tested		133.540	**<0.001**	Not tested		Not tested	
Age	Not tested		Not tested		Not tested		Not tested		3.231	0.072	Not tested	
Breeding x sex	Not tested		Not tested		Not tested		1.347	0.246	Not tested		Not tested	
Breeding x age	Not tested		Not tested		Not tested		Not tested		0.893	0.345	Not tested	
Mating treatment	Not tested		Not tested		Not tested		Not tested		Not tested		203.700	**<0.001**
**Random effects:**	**Variance**		**Variance**		**Variance**		**Variance**		**Variance**		**Variance**	
Petri dish	0.000		Not tested		Not tested		Not tested		Not tested		Not tested	
Family in P‐generation	0.000		0.046		0.353		0.004		0.018		0.009	

Note

The table gives *p* and *X²*‐Values from chi‐square likelihood ratio tests for all fixed effects in the respective (G)LMM as well as the amount of variance explained by the random effects in each model (extracted from the minimal adequate mixed effects models). Significant *p*‐Values are indicated in bold.

**Table 2:** Extent of inbreeding depression in the fitness‐related traits development time, larval and adult survival rate, female and male body mass, egg number (experiment 1), and hatching rate (experiment 2, the mating treatments N/N and S/S were used) of *Phaedon cochleariae*

Trait	δ
Development time	0.066
Larval survival	0.404
Adult survival	0.189
Body mass females	0.077
Body mass males	0.007
Egg number	0.394
Larval hatching rate	0.254

*Note*

The coefficient of inbreeding depression (δ = $({\overset{¯}{\omega }}_{\text{Outbred}}-{\overset{¯}{\omega }}_{\text{Inbred}}/$ ${\overset{¯}{\omega }}_{\text{Outbred}})$ was calculated according to Hedrick and Kalinowski (2000). The average over all families is shown.

### 3.2 Effects of nonsibling *versus* sibling male mating partner order on larval hatching rate

**Figure 3:**
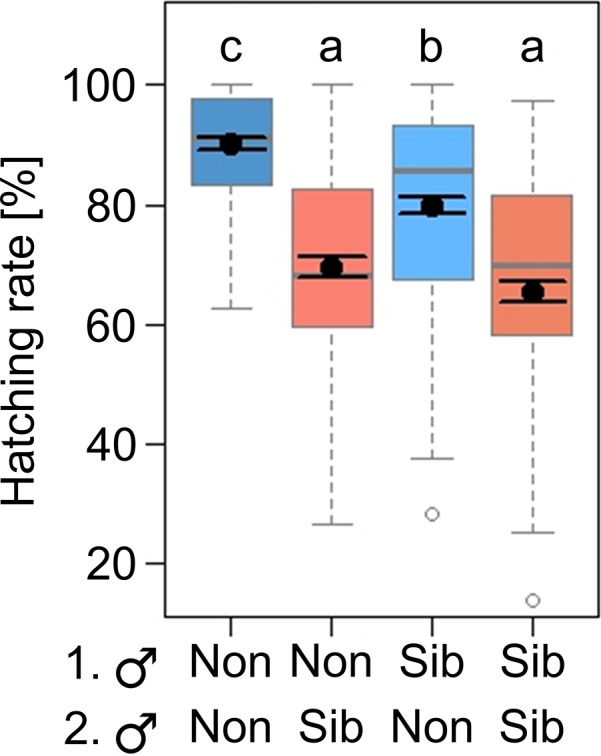
Effects of the mating treatment [male order: nonsibling, nonsibling (Non/Non) *versus* nonsibling, sibling (Non/Sib) *versus* sibling, nonsibling (Sib/Non) *versus* sibling, sibling (Sib/Sib)] on the larval hatching rate of eggs laid per female of *Phaedon cochleariae* (*N* = 24–25). The box‐whisker plots show the following statistics: medians (solid gray lines), interquartile range (boxes), 1.5 * lower/upper quartile (whiskers), outliers (white dots), and least square means with their standard error (black dots with error bars) extracted from the minimal adequate (G)LMM. The letters indicate significant differences between the treatment groups and are based on a Tukey post hoc test on the minimal adequate GLMM

All female individuals mated with the first and the second male partner during experiment 2. The hatching rate of larvae descending from these matings was significantly affected by the mating treatment, which combined different breeding levels (outbreeding *versus* inbreeding) and a different succession of the two breeding levels in a sequential mating with different male partners (Figure 3, Tables 1, 2). Larvae descending from females mated with two nonsibling males had the highest hatching rate (90.1%) and larvae descending from females either mated with two siblings (65.4%) or first with a nonsibling and consecutively with a sibling (69.4%) had the lowest hatching rate. Larvae descending from eggs laid by females, which were first mated with a sibling and consecutively with a nonsibling, had an intermediate hatching rate (79.9%).

## 4 DISCUSSION

### 4.1 Lifetime inbreeding depression in *P*. *cochleariae*

The present study revealed that mustard leaf beetles suffer from strong inbreeding depression throughout their ontogeny. Inbreeding does not only reduce the larval hatching rate of the beetle as detected in a previous study (Müller & Müller, 2016), but also crucially increases the mortality of larvae prior adult emergence and of young adults. Moreover, the reproductive output of inbred females was reduced compared to outbred females, although their body mass was not decreased by inbreeding. Comparable effects were previously detected in the beetle *Propylea quatuordecimpunctata* (Morjan, Obrycki, & Krafsur, 1999), which suggests that body mass might be more robust against inbreeding impacts than other fitness‐related traits. Possible effects of the males’ breeding status on the reproductive success remain to be tested in *P. cochleariae*. Given the comprehensive detrimental effects of inbreeding detected in this and in previous studies on *P. cochleariae* (Müller & Juškauskas, 2018; Müller & Müller, 2016), it can be expected that the reproduction abilities of inbred males are lower relative to outbred males. Other studies illustrate that sperm competiveness and/or amount are depressed by inbreeding in other arthropod species (Ala‐Honkola et al., 2013; Fox, Xu, Wallin, & Curtis, 2012; Konior, Keller, & Radwan, 2005; Michalczyk, Martin, Millard, Emerson, & Gage, 2010).

In summary, our results for lifetime inbreeding depression in *P. cochleariae* suggest that natural populations of the species, in which inbreeding depression is also detectable (Müller & Juškauskas, 2018), have a risk to become extinct within a few generations. This scenario can be relevant for a wide range of species that suffer from strong inbreeding depression after only one or few generations of nonrandom mating (Bilde et al., 2007; Fox et al., 2007; Roff, 1998). This applies as long as the purging of deleterious recessive mutations does not mitigate or eliminate inbreeding depression under nonrandom mating conditions (Crnokrak & Barrett, 2002; Hedrick & Garcia‐Dorado, 2016). In addition, inbreeding effects on the behavioral phenotype, that is, personality, might either amplify or counteract the reduced viability of inbred *P. cochleariae* individuals, depending on the environment (Müller & Juškauskas, 2018). Such inbreeding by environment interaction effects on the behavioral phenotype represents a so far underestimated field of evolutionary ecology and might be relevant over a wide range of animal species (Ala‐Honkola, Uddström, Diaz Pauli, & Lindström, 2009; Aspi, 2000; Briskie & Mackintosh, 2004). Taken together, our studies on the inbreeding effects on lifetime fitness and the behavior in *P. chochleariae* depict a comprehensive scenario of possible costs associated with inbreeding in insect populations. Such laboratory studies are a first important step to achieve a better understanding of the manifold inbreeding effects suffered in wild populations. However, the extent of inbreeding depression under benign laboratory conditions may not precisely predict the magnitude of inbreeding depression under more stressful field conditions in natural populations (Armbruster & Reed, 2005). Some studies illustrated that results gained from laboratory‐reared populations can predict the performance of wild populations under field conditions (Coelho, Rugman‐Jones, Reigada, Stouthamer, & Parra, 2016; Fox et al., 2007). Nevertheless, there is broad evidence that the magnitude of inbreeding depression is higher under stressful field conditions relative to benign laboratory conditions (Fox & Reed, 2011). As global change not only increases inbreeding rates through habitat loss and fragmentation, but simultaneously raises the levels of abiotic and biotic stress in remaining habitats (Davis, 2003; Kingslover, Diamond, & Buckley, 2013), inbreeding depression is a huge recent and future challenge for many species (Andersen, Fog, & Damgaard, 2004).

### 4.2 Evidence for second male sperm precedence in *P. cochleariae*

In our study, no female‐mediated sibling sperm discrimination was detectable in *P. cochleariae*. If postcopulatory inbreeding avoidance mechanisms would exist, one would expect a similar larval hatching rate resulting from a mating with a nonsibling and a sibling male in both orders, because females might be able to preferentially choose sperm of nonsiblings. This phenomenon is postulated as cryptic female choice (Eberhard, 1996) and is supposed to be realized in various arthropod species (Fedina, 2007; Peretti & Eberhard, 2010). However, in the present study, it was not detected. Contrary, the hatching rate resulting from mating with a sibling followed by a nonsibling was significantly higher compared to a mating in the opposite order. Moreover, the hatching rate was highest in the mating with two nonsiblings. This points to second male sperm precedence, a phenomenon in polyandrous species in which the second/last male mating partner sires more offspring than the first (Kehl, Karl, & Fischer, 2013; Xu & Wang, 2010). Paternity analyses must be conducted to clarify this assumption in *P. cochleariae*. If the break between the first male and second male during the sequential mating would be shorter than the applied 18 hr, the impact of the second male may become even more pronounced than it already was. A potential mechanism behind second male sperm precedence can be associated with either females or males. Females can release sperm of the first mating from their reproductive tract (Snook & Hosken, 2004). Alternatively, the second male might displace the sperm of the first male (Xu & Wang, 2010). The realized mechanisms underlying second male sperm precedence in *P. cochleariae* remain to be clarified in future studies. Second male sperm precedence would shed a new light on a specific behavior previously observed in *P. cochleariae*. Couples of the beetle often mate several hours. During that time, no other male has the possibility to mate with the female. This long mating duration is potentially a mate guarding behavior or, more precisely, a mounting behavior practiced by males. In doing so, males may prevent fertilization by other males to improve their own paternity chances, which was also observed in the West Indian sweetpotato weevil (Sato & Kohama, 2007). A mate guarding behavior combined with second male sperm precedence and a possibility of remating, can enhance male fitness (Arnqvist, 1988; Baxter, Barnett, & Dukas, 2015; Calbacho‐Rosa, Cordoba‐Aguilar, & Peretti, 2010).

Concluding from a previous (Müller & Müller, 2016) and the present study, neither pre‐ nor postcopulatory inbreeding avoidance mechanisms could be detected in *P. cochleariae*. This is surprising because *P. cochleariae* suffers from severe inbreeding depression in a laboratory (this study) and a wild population (Müller & Juškauskas, 2018). Simultaneously, these beetles have distinct family‐specific CHC patterns, which could function as recognition cue for inbreeding avoidance. However, the absence of inbreeding avoidance in spite of the expression of inbreeding depression was also observed for burying beetles (Mattey & Smiseth, 2015; Pilakouta & Smiseth, 2016). Hence, the question arises whether any other mechanisms to avoid inbreeding costs exist in these species. One possibility could be a specific dispersal behavior, which was detected in many species throughout the animal kingdom (Pusey & Wolf, 1996). Indeed, in a previous study, we found that wild *P. cochleariae* males are more active than females and cover longer distances (Müller & Juškauskas, 2018). This result suggests that the dispersal of males may avoid the mating among close relatives in natural populations of the species. Sex‐specific dispersal can be observed across a wide range of animal species and is often expressed to avoid inbreeding and/or intraspecific competition (Pusey, 1987; Pusey & Wolf, 1996). Which sex disperses is determined by the ratio of costs (increased predation or less mates in the new habitat) and benefits (less competition and relatives as mates) that are associated with dispersal (Motro, 1991; Perrin & Mazalov, 1999; Yoder, Marschall, & Swanson, 2004).

Moreover, a polyandrous mating system might exist in *P. cochleariae*. As postulated by the fertility restoration hypothesis (Bayoumy et al., 2015), such mating systems can evolve in species, if the reproductive success of females is decreased by inbreeding, but increased by outbreeding. A further mating assay, in which females have the possibility to mate with single nonsiblings or siblings or with groups of either nonsiblings, siblings or a mixed group of nonsiblings and siblings, can clarify if polyandry is beneficial for *P. cochleariae* in the context of inbreeding. Females of polyandrous species may use CHC patterns and a CHC based chemosensory self‐referencing mechanisms to increase the diversity of their mating partners (Weddle, Hunt, et al., 2013; Weddle, Steiger, et al., 2013) and consequently the genetic diversity of their offspring. However, even for polyandrous species, an increasing degree of relatedness in a given population following habitat loss and fragmentation will decrease the efficiency of inbreeding avoidance by multiple paternity.

Overall, the present study highlights the need to measure inbreeding depression throughout the entire ontogeny of a study organism. Focusing on single traits or restricted periods in ontogeny could potentially lead to an under‐estimation of the inbreeding effects on fitness‐related traits. Although we found pronounced lifetime inbreeding depression, a postcopulatory inbreeding avoidance mechanism was not detected, which supports the existence of a polyandrous mating system or specific dispersal strategies for inbreeding avoidance. Instead, we disclosed evidence for second male sperm precedence, which can potentially explain specific behavioral traits of our study species, that is, a mounting behavior of males after mating.

## CONFLICT OF INTEREST

None declared.

## AUTHOR CONTRIBUTIONS

TM designed the study. TDL and TM conducted the breeding experiment and the bioassays and collected the data. KS and TM analyzed the data and prepared the figures. TM and KS wrote and revised the manuscript.

## References

[ece34205-bib-0001] Agnarsson, I. , Aviles, L. , & Maddison, W. P. (2013). Loss of genetic variability in social spiders: Genetic and phylogenetic consequences of population subdivision and inbreeding. Journal of Evolutionary Biology, 26, 27–37. 10.1111/jeb.12022 23145542 PMC3588177

[ece34205-bib-0002] Aguilar, R. , Quesada, M. , Ashworth, L. , Herrerias‐Diego, Y. , & Lobo, J. (2008). Genetic consequences of habitat fragmentation in plant populations: Susceptible signals in plant traits and methodological approaches. Molecular Ecology, 17, 5177–5188. 10.1111/j.1365-294X.2008.03971.x 19120995

[ece34205-bib-0003] Ala‐Honkola, O. , Hosken, D. J. , Manier, M. K. , Lupold, S. , Droge‐Young, E. M. , Berben, K. S. , … Pitnick, S. (2013). Inbreeding reveals mode of past selection on male reproductive characters in *Drosophila melanogaster* . Ecology and Evolution, 3, 2089–2102. 10.1002/ece3.625 23919154 PMC3728949

[ece34205-bib-0004] Ala‐Honkola, O. , Uddström, A. , Diaz Pauli, B. , & Lindström, K. (2009). Strong inbreeding depression in male mating behaviour in a poeciliid fish. Journal of Evolutionary Biology, 22, 1396–1406. 10.1111/j.1420-9101.2009.01765.x 19486236

[ece34205-bib-0005] Andersen, L. W. , Fog, K. , & Damgaard, C. (2004). Habitat fragmentation causes bottlenecks and inbreeding in the European tree frog (*Hyla arborea*). Proceedings of the Royal Society of London, Series B: Biological Sciences, 271, 1293–1302. 10.1098/rspb.2004.2720 15306354 PMC1691722

[ece34205-bib-0006] Armbruster, P. , & Reed, D. H. (2005). Inbreeding depression in benign and stressful environments. Heredity, 95, 235–242. 10.1038/sj.hdy.6800721 16077737

[ece34205-bib-0007] Arnqvist, G. (1988). Mate guarding and sperm displacement in the water strider *Gerris lateralis* Schumm. (Heteroptera: Gerridae). Freshwater Biology, 19, 269–274. 10.1111/j.1365-2427.1988.tb00347.x

[ece34205-bib-0008] Aspi, J. (2000). Inbreeding and outbreeding depression in male courtship song characters in *Drosophila montana* . Heredity, 84, 273–282. 10.1046/j.1365-2540.2000.00655.x 10762398

[ece34205-bib-0009] Banks, S. C. , Piggott, M. P. , Stow, A. J. , & Taylor, A. C. (2007). Sex and sociality in a disconnected world: A review of the impacts of habitat fragmentation on animal social interactions. Canadian Journal of Zoology, 85, 1065–1079. 10.1139/Z07-094

[ece34205-bib-0010] Bates, D. , Maechler, M. , Bolker, B. , & Walker, S. (2014). Linear mixed‐effects models using Eigen and S4. R package version 1.1.12. Retrieved from http://cran.R-project.org/package=lme4

[ece34205-bib-0011] Bates, A. J. , Sadler, J. P. , Grundy, D. , Lowe, N. , Davis, G. , Baker, D. , … Young, H. (2014). Garden and landscape‐scale correlates of moths of differing conservation status: Significant effects of urbanization and habitat diversity. PLoS One, 9, e86925 10.1371/journal.pone.0086925 24475197 PMC3903603

[ece34205-bib-0012] Baxter, C. M. , Barnett, R. , & Dukas, R. (2015). Aggression, mate guarding and fitness in male fruit flies. Animal Behavior, 109, 235–241. 10.1016/j.anbehav.2015.08.023

[ece34205-bib-0013] Bayoumy, M. H. , Michaud, J. P. , & Bain, C. (2015). Polyandry restores female fertility and paternal effects diminished by inbreeding in *Hippodamia convergens* . Ecological Entomology, 40, 596–602. 10.1111/een.12230

[ece34205-bib-0014] Bilde, T. , Maklakov, A. A. , & Schilling, N. (2007). Inbreeding avoidance in spiders: Evidence for rescue effect in fecundity of female spiders with outbreeding opportunity. Journal of Evolutionary Biology, 20, 1237–1242. 10.1111/j.1420-9101.2006.01280.x 17465934

[ece34205-bib-0015] Bouchebti, S. , Durier, V. , Pasquaretta, C. , Rivault, C. , & Lihoreau, M. (2016). Subsocial cockroaches *Nauphoeta cinerea* mate indiscriminately with kin despite high costs of inbreeding. PLoS One, 11, e0162548 10.1371/journal.pone.0162548 27655156 PMC5031396

[ece34205-bib-0016] Bretman, A. , Newcombe, D. , & Tregenza, T. (2009). Promiscuous females avoid inbreeding by controlling sperm storage. Molecular Ecology, 18, 3340–3345. 10.1111/j.1365-294X.2009.04301.x 19694961

[ece34205-bib-0017] Briskie, J. V. , & Mackintosh, M. (2004). Hatching failure increases with severity of population bottleneck in birds. Proceedings of the National Academy of Sciences of the United States of America, 2, 558–561. 10.1073/pnas.0305103101 PMC327186 14699045

[ece34205-bib-0018] Calbacho‐Rosa, L. , Cordoba‐Aguilar, A. , & Peretti, A. V. (2010). Occurrence and duration of post‐copulatory mate guarding in a spider with last sperm precedence. Behaviour, 147, 1267–1283. 10.1163/000579510X514544

[ece34205-bib-0019] Charlesworth, D. , & Willis, J. H. (2009). The genetics of inbreeding depression. Nature Reviews Genetics, 10, 783–796. 10.1038/nrg2664 19834483

[ece34205-bib-0020] Coelho, A. , Rugman‐Jones, P. F. , Reigada, C. , Stouthamer, R. , & Parra, J. R. P. (2016). Laboratory performance predicts the success of field releases in inbred lines of the egg parasitoid *Trichogramma pretiosum* (Hymenoptera: Trichogrammatidae). PLoS ONS, 11, e0146153 10.1371/journal.pone.0146153 PMC4701434 26730735

[ece34205-bib-0021] Crnokrak, P. , & Barrett, S. C. H. (2002). Perspective: Purging the genetic load: A review of the experimental evidence. Evolution, 56, 2347–2358. 10.1111/j.0014-3820.2002.tb00160.x 12583575

[ece34205-bib-0022] Davis, A. D. (2003). Biotic globalization: Does competition from introduced species threaten biodiversity? BioScience, 53, 481–489. 10.1641/0006-3568(2003)053[0481:BGDCFI]2.0.CO;2

[ece34205-bib-0023] Duthie, A. B. , Bocedi, G. , & Reid, J. M. (2016). When does female multiple mating evolve to adjust inbreeding? Effects of inbreeding depression, direct costs, mating constraints, and polyandry as a threshold trait. Evolution, 70, 1927–1943. 10.1111/evo.13005 27464756 PMC5053304

[ece34205-bib-0024] Eberhard, W. G. (1996). Female control: Sexual selection by cryptic female choice. Princeton: Princeton University Press.

[ece34205-bib-0025] Edvardsson, M. , Rodriguez‐Munoz, R. , & Tregenza, T. (2008). No evidence that female bruchid beetles, *Callosobruchus maculatus*, use remating to reduce costs of inbreeding. Animal Behavior, 75, 1519–1524. 10.1016/j.anbehav.2007.10.005

[ece34205-bib-0026] Fedina, T. Y. (2007). Cryptic female choice during spermatophore transfer in *Tribolium castaneum* (Coleoptera: Tenebrionidae). Journal of Insect Physiology, 53, 93–98. 10.1016/j.jinsphys.2006.10.011 17161846

[ece34205-bib-0027] Fox, C. W. , & Reed, D. H. (2011). Inbreeding depression increases with environmental stress: An experimental study and meta‐analysis. Evolution, 97, 49–54. 10.1111/j.1558-5646.2010.01108.x 20731715

[ece34205-bib-0028] Fox, C. W. , Scheibly, K. L. , Smith, B. P. , & Wallin, W. G. (2007). Inbreeding depression in two seed‐feeding beetles, *Callosobruchus maculatus* and *Stator limbatus* (Coleoptera: Chrysomelidae). Bulletin of Entomological Research, 97, 49–54. 10.1017/S0007485307004737 17298681

[ece34205-bib-0029] Fox, C. W. , Xu, J. , Wallin, W. G. , & Curtis, C. L. (2012). Male inbreeding status affects female fitness in a seed‐feeding beetle. Journal of Evolutionary Biology, 25, 29–37. 10.1111/j.1420-9101.2011.02400.x 21995954

[ece34205-bib-0030] Geiselhardt, S. , Otte, T. , & Hilker, M. (2012). Looking for a similar partner: Host plants shape mating preferences of herbivorous insects by altering their contact pheromones. Ecology Letters, 15, 971–977. 10.1111/j.1461-0248.2012.01816.x 22708843

[ece34205-bib-0031] Haddad, N. M. , Brudvig, L. A. , Clobert, J. , Davie, K. F. , Gonzalez, A. , Holt, R. D. , … Townshend, J. R. (2015). Habitat fragmentation and its lasting impact on Earth's ecosystems. Science Advances, 2, e1500052. 10.1126/sciadv.1500052 PMC4643828 26601154

[ece34205-bib-0032] Harano, T. (2011). Inbreeding depression in development, survival, and reproduction in the adzuki bean beetle (*Callosobruchus chinensis*). Ecological Research, 26, 327–332. 10.1007/s11284-010-0787-y

[ece34205-bib-0033] Hedrick, P. W. , & Garcia‐Dorado, A. (2016). Understanding inbreeding depression, purging, and genetic rescue. Trends in Ecology & Evolution, 31, 940–952. 10.1016/j.tree.2016.09.005 27743611

[ece34205-bib-0034] Hedrick, P. W. , & Kalinowski, S. T. (2000). Inbreeding depression in conservation biology. Annual Review of Ecology and Systematics, 31, 139–162. 10.1146/annurev.ecolsys.31.1.139

[ece34205-bib-0035] Herzner, G. , Schmitt, T. , Heckel, F. , Schreier, P. , & Strohm, E. (2006). Brothers smell similar: Variation in the sex pheromone of male european beewolves *Philanthus triangulum* F. (Hymenoptera: Crabronidae) and its implications for inbreeding avoidance. Biological Journal of the Linnean Society, 89, 433–442. 10.1111/j.1095-8312.2006.00684.x

[ece34205-bib-0036] Hothorn, T. , Bretz, F. , & Westfall, P. (2008). Simultaneous inference in general parametric models. Biometrical Journal, 50, 346–363. R package version 1.4.8. Retrieved from http://cran.p-project.org/package=multcomp 18481363 10.1002/bimj.200810425

[ece34205-bib-0037] Howard, R. W. , & Blomquist, G. J. (2005). Ecological, behavioral, and biochemical aspects of insect hydrocarbons. Annual Review of Entomology, 50, 371–393. 10.1146/annurev.ento.50.071803.130359 15355247

[ece34205-bib-0038] Kehl, T. , Karl, I. , & Fischer, K. (2013). Old‐male paternity advantage is a function of accumulating sperm and last‐male precedence in a butterfly. Molecular Ecology, 22, 4289–4297. 10.1111/mec.12386 23889582

[ece34205-bib-0039] Keller, L. F. , & Waller, D. M. (2002). Inbreeding effects in wild populations. Trends in Ecology & Evolution, 17, 230–241. 10.1016/S0169-5347(02)02489-8

[ece34205-bib-0040] Kingslover, J. G. , Diamond, S. E. , & Buckley, L. B. (2013). Heat stress and the fitness consequences of climate change for terrestrial ectotherms. Functional Ecology, 27, 1415–1423. 10.1111/1365-2435.12145

[ece34205-bib-0041] Kokko, H. , & Ots, I. (2006). When not to avoid inbreeding. Evolution, 60, 467–475. 10.1111/j.0014-3820.2006.tb01128.x 16637492

[ece34205-bib-0042] Konior, M. , Keller, L. , & Radwan, J. (2005). Effect of inbreeding and heritability of sperm competition success in the bulb mite *Rhizoglyphus robini* . Heredity, 94, 577–581. 10.1038/sj.hdy.6800649 15742000

[ece34205-bib-0043] Lane, J. E. , Forrest, M. N. K. , & Willis, C. K. R. (2011). Anthropogenic influences on natural animal mating systems. Animal Behavior, 81, 909–917. 10.1016/j.anbehav.2011.02.003

[ece34205-bib-0044] Lenth, R. V. (2016). Least square means: The R package lsmeans. Journal of Statistical Software, 69, 1–33. R‐package version 2.26.3. Retrieved from http://cran.R-project.org/package=lsmeans

[ece34205-bib-0045] Lewis, Z. , & Wedell, N. (2009). Male moths reduce sperm investment in relatives. Animal Behavior, 77, 1547–1550. 10.1016/j.anbehav.2009.03.013

[ece34205-bib-0046] Lihoreau, M. , & Rivault, C. (2009). Kin recognition via cuticular hydrocarbons shapes cockroach social life. Behavioral Ecology, 20, 46–53. 10.1093/beheco/arn113

[ece34205-bib-0047] Lihoreau, M. , Zimmer, C. , & Rivault, C. (2007). Kin recognition and incest avoidance in a group‐living insect. Behavioral Ecology, 18, 880–887. 10.1093/beheco/arm046

[ece34205-bib-0048] Liu, X. , Tu, X. , He, H. , Chen, C. , & Xue, F. (2014). Evidence for inbreeding depression and pre‐copulatory, but not post copulatory inbreeding avoidance in the cabbage beetle *Colaphellus bowringi* . PLoS One, 9, e94389 10.1371/journal.pone.0094389 24718627 PMC3981785

[ece34205-bib-0049] Mattey, S. N. , & Smiseth, P. T. (2015). No inbreeding avoidance by female burying beetles regardless of whether they encounter males simultaneously or sequentially. Ethology, 121, 1031–1038. 10.1111/eth.12417

[ece34205-bib-0050] Menzel, F. , Radke, R. , & Foitzik, S. (2016). Odor diversity decreases with inbreeding in the ant *Hypoponera opacior* . Evolution, 70, 2573–2582. 10.1111/evo.13068 27641363

[ece34205-bib-0051] Metzger, M. , Bernstein, C. , Hoffmeister, T. S. , & Desouhant, E. (2010). Does kin recognition and sib‐mating avoidance limit the risk of genetic incompatibility in a parasitic wasp? PLoS One, 5, e13505 10.1371/journal.pone.0013505 20976063 PMC2957437

[ece34205-bib-0052] Michalczyk, Ł. , Martin, O. Y. , Millard, A. L. , Emerson, B. C. , & Gage, M. J. (2010). Inbreeding depresses sperm competitiveness, but not fertilization or mating success in male *Tribolium castaneum* . Proceedings of the Royal Society of London, Series B: Biological Sciences, 277, 3483–3491. 10.1098/rspb.2010.0514 20554548 PMC2982220

[ece34205-bib-0053] Morjan, W. E. , Obrycki, J. J. , & Krafsur, E. S. (1999). Inbreeding effects on *Propylea quatuordecimpunctata* (Coleoptera: Coccinellidae). Annals of the Entomological Society of America, 92, 260–268. 10.1093/aesa/92.2.260

[ece34205-bib-0054] Motro, U. (1991). Avoiding inbreeding and sibling competition: The evolution of sexual dimorphism for dispersal. American Naturalist, 137, 108–115. 10.1086/285148

[ece34205-bib-0055] Müller, T. , & Juškauskas, A. (2018). Inbreeding affects personality and fitness of a leaf beetle. Animal Behavior, 138, 29–37. 10.1016/j.anbehav.2018.02.002

[ece34205-bib-0056] Müller, T. , & Müller, C. (2016). Consequences of mating with siblings and nonsiblings on the reproductive success in a leaf beetle. Ecology and Evolution, 6, 3185–3197. 10.1002/ece3.2103 27103986 PMC4829044

[ece34205-bib-0057] Murphy, S. M. , Battocletti, A. H. , Tinghitella, R. M. , Wimp, G. M. , & Ries, L. (2016). Complex community and evolutionary responses to habitat fragmentation and habitat edges: What can we learn from insect science? Current Opinion in Insect Science, 14, 61–65. 10.1016/j.cois.2016.01.007 27436648

[ece34205-bib-0058] Peer, K. , & Taborsky, M. (2005). Outbreeding depression, but no inbreeding depression in haplodiploid Ambrosia beetles with regular sibling mating. Evolution, 59, 317–323. 10.1111/j.0014-3820.2005.tb00992.x 15807418

[ece34205-bib-0059] Peretti, A. V. , & Eberhard, W. G. (2010). Cryptic female choice via sperm dumping favours male copulatory courtship in a spider. Journal of Evolutionary Biology, 23, 271–281. 10.1111/j.1420-9101.2009.01900.x 20487130

[ece34205-bib-0060] Perrin, N. , & Mazalov, V. (1999). Dispersal and inbreeding avoidance. American Naturalist, 154, 282–292. 10.1086/303236 10506544

[ece34205-bib-0061] Pilakouta, N. , & Smiseth, P. T. (2016). Maternal effects alter the severity of inbreeding depression in the offspring. Proceedings of the Royal Society of London, Series B: Biological Sciences, 283, 20161023 10.1098/rspb.2016.1023 27629026 PMC5031652

[ece34205-bib-0062] Pilakouta, N. , & Smiseth, P. T. (2017). Female mating preferences for outbred *versus* inbred males are conditional upon the female's own inbreeding status. Animal Behavior, 123, 369–374. 10.1016/j.anbehav.2016.11.023

[ece34205-bib-0063] Pusey, A. E. (1987). Sex‐biased dispersal and inbreeding avoidance in birds and mammals. Trends in Ecology & Evolution, 2, 295–299. 10.1016/0169-5347(87)90081-4 21227869

[ece34205-bib-0064] Pusey, A. , & Wolf, M. (1996). Inbreeding avoidance in animals. Trends in Ecology & Evolution, 11, 201–206. 10.1016/0169-5347(96)10028-8 21237809

[ece34205-bib-0065] R Development Core Team (2015). R: A language and environment for statistical computing. Version 3.2.3. Retrieved from http://cran.R-project.org/

[ece34205-bib-0066] Richardson, J. , & Smiseth, P. T. (2017). Intraspecific competition and inbreeding depression: Increased competitive effort by inbred males is costly to outbred opponents. American Naturalist, 189, 539–548. 10.1086/691328 28410022

[ece34205-bib-0067] Roff, D. A. (1998). Effects of inbreeding on morphological and life history traits of the sand cricket, *Gryllus firmus* . Heredity, 81, 28–37. 10.1046/j.1365-2540.1998.00363.x

[ece34205-bib-0068] Sato, Y. , & Kohama, T. (2007). Post‐copulatory mounting behavior of the West Indian sweetpotato weevil, *Euscepes postfasciatus* (Fairmaire) (Coleoptera: Curculionidae). Ethology, 113, 183–189. 10.1111/j.1439-0310.2006.01309.x

[ece34205-bib-0069] Schmitz, C. , van Meijl, H. , Kyle, P. , Nelson, G. C. , Fujimori, S. , Gurgel, A. , … Valin, H. (2014). Land‐use change trajectories up to 2050: Insights from a global agro‐ economic model comparison. Agricultural Economics, 45, 69–84. 10.1111/agec.12090

[ece34205-bib-0070] Simmons, L. W. , Beveridge, M. , Wedell, N. , & Tregenza, T. (2006). Postcopulatory inbreeding avoidance by female crickets only revealed by molecular markers. Molecular Ecology, 15, 3817–3824. 10.1111/j.1365-294X.2006.03035.x 17032276

[ece34205-bib-0071] Snook, R. R. , & Hosken, D. J. (2004). Sperm death and dumping in *Drosophila* . Nature, 428, 939–941. 10.1038/nature02455 15118726

[ece34205-bib-0072] Swift, T. L. , & Hannon, S. J. (2010). Critical threshold associated with habitat loss: A review of the concepts, evidence, and applications. Biological Reviews, 85, 35–53. 10.1111/j.1469-185X.2009.00093.x 19930172

[ece34205-bib-0073] Thomas, M. L. , & Simmons, L. W. (2011). Crickets detect the genetic similarity of mating partners via cuticular hydrocarbons. Journal of Evolutionary Biology, 24, 1793–1800. 10.1111/j.1420-9101.2011.02319.x 21649764

[ece34205-bib-0074] Tregenza, T. , & Wedell, N. (2002). Polyandrous females avoid costs of inbreeding. Nature, 415, 71–73. 10.1038/415071a 11780118

[ece34205-bib-0075] Venables, W. N. , & Ripley, B. D. (2000). Modern Applied Statistics with S, 4th ed. New York, USA: Springer R‐package version 7.3.45. Retrieved from http://CRAN.R-project.org/package=MASS

[ece34205-bib-0076] Weddle, C. B. , Hunt, J. , & Sakaluk, S. K. (2013). Self‐referent phenotype matching and its role in female mate choice in arthropods. Current Zoology, 59, 239–248. 10.1093/czoolo/59.2.239

[ece34205-bib-0077] Weddle, C. B. , Steiger, S. , Hamaker, C. G. , Ower, G. D. , Mitchell, C. , Sakaluk, S. K. , & Hunt, J. (2013). Cuticular hydrocarbons as a basis for chemosensory self‐referencing in crickets: A potentially universal mechanism facilitating polyandry in insects. Ecology Letters, 16, 346–353. 10.1111/ele.12046 23279570

[ece34205-bib-0078] Welke, K. , & Schneider, J. M. (2009). Inbreeding avoidance through cryptic female choice in the cannibalistic orb‐web spider *Argiope lobata* . Behavioral Ecology, 20, 1056–1062. 10.1093/beheco/arp097

[ece34205-bib-0079] Whitehorn, P. R. , Tinsley, M. C. , & Goulson, D. (2009). Kin recognition and inbreeding reluctance in bumblebees. Apidologie, 40, 627–633. 10.1051/apido/2009050

[ece34205-bib-0080] Xu, J. , & Wang, Q. (2010). Mechanisms of last male precedence in a moth: Sperm displacement at ejaculation and storage sites. Behavioral Ecology, 21, 714–721. 10.1093/beheco/arq044

[ece34205-bib-0081] Yoder, J. M. , Marschall, E. A. , & Swanson, D. A. (2004). The cost of dispersal: Predation as a function of movement and site familiarity in ruffed grouse. Behavioral Ecology, 15, 469–476. 10.1093/beheco/arh037

